# Cost-effectiveness of brentuximab vedotin compared with conventional chemotherapy for relapsed or refractory classic Hodgkin lymphoma in China

**DOI:** 10.1186/s13561-024-00514-6

**Published:** 2024-06-06

**Authors:** Shitong Xie, Yanan Sheng, Ling-Hsiang Chuang, Jing Wu

**Affiliations:** 1 School of Pharmaceutical Science and Technology, Faculty of Medicine, Tianjin University, Tianjin, China; 2 Center for Social Science Survey and Data, Tianjin University, Tianjin, China; 3 Medical Affairs, Takeda (China) International Trading Company, Beijing, China; 4 Umea University, Umea, Sweden

**Keywords:** Relapsed or refractory classic Hodgkin lymphoma, Brentuximab vedotin, Cost-effectiveness analysis, Cost-utility analysis, Chemotherapy, China

## Abstract

**Background:**

Relapsed or refractory classic Hodgkin lymphoma (RRcHL) associates with poor prognosis and heavy disease burden to patients. This study evaluated the cost-effectiveness of brentuximab vedotin (BV) in comparison to conventional chemotherapy in patients with RRcHL, from a Chinese healthcare perspective.

**Methods:**

The lifetime cost and quality adjusted life years (QALYs) were estimated through a partitioned survival model with three health states (progression free, post progression, and death). Two cohorts for each BV arm and chemotherapy arm were built, representing patients with and without transplant after BV or chemotherapy, respectively. Clinical parameters were retrieved from BV trials and the literature. Resource utilization data were mainly collected from local expert surveys and cost parameters were reflecting local unit prices. Utility values were sourced from the literature. A discount rate of 5% was employed according to the Chinese guideline. A series of deterministic and probabilistic sensitivity analyses were conducted to evaluate the robustness and uncertainty associated with the model.

**Results:**

Results of the base case analysis showed that the incremental cost-effectiveness ratio (ICER) for BV versus chemotherapy was $2,867 (¥19,774). The main model driver was the superior progression-free and overall survival benefits of BV. The ICERs were relatively robust in a series of sensitivity analyses, all under a conventional decision threshold (1 time of Chinese per capita GDP). With this conventional threshold, the probability of BV being cost-effective was 100%.

**Conclusions:**

Brentuximab vedotin can be considered a cost-effective treatment versus conventional chemotherapy in treating relapsed or refractory classic Hodgkin lymphoma in China.

**Supplementary Information:**

The online version contains supplementary material available at 10.1186/s13561-024-00514-6.

## Highlights

In China, BV was approved in 2020 for the treatment of adult patients with CD30-positive RRcHL. However, the economic value of BV compared with conventional therapy remains unknown.

This study evaluated the cost-effectiveness of brentuximab vedotin (BV) in comparison to conventional chemotherapy in patients with RRcHL, from a Chinese healthcare perspective.

Brentuximab vedotin can be considered a cost-effective treatment versus conventional chemotherapy in treating relapsed or refractory classic Hodgkin lymphoma in China, with the ICER under a conventional decision threshold (1 time of Chinese per capita GDP).

**Supplementary Information:**

The online version contains supplementary material available at 10.1186/s13561-024-00514-6.

## Introduction

Hodgkin Lymphoma (HL) is a rare type of hematologic cancer, that accounts for 10–20% of all lymphomas, mainly involving lymph nodes and lymphatic system [1]. Classic Hodgkin Lymphoma (cHL) accounts for approximately 95% of all Hodgkin lymphomas [1], with unique histopathologic features of malignant Hodgkin Reed-Sternberg (HRS) cells in an inflammatory background. Globally, a total of 83,000 new cases of HL and 23,000 deaths from HL were estimated in 2020 [2]. In China the numbers were 6,829 reported new cases and 2,807 deaths [3]. The incidence peak occurs in adults aged around 40 years with a slight male predominance (0.5 men vs. 0.3 women per 100,000) [3]. According to the 2017 China Cancer Registry Annual Report, the age-standardized mortality rate of HL is 0.13/100,000 [4].

In the past 40 years, the 10-year survival of patients with HL has increased from 47 to 80% [5]; after initial treatment, chemotherapy with or without radiotherapy, most patients can be potentially cured [5]. Nevertheless, about 10-30% of patients relapse of experience refractoriness after first-line chemotherapy [6]. The standard therapy for relapsed or refractory patients is high-dose salvage chemotherapy followed by autologous stem cell transplantation (ASCT); the cure rate is about 50% [1]. The prognosis of patients who failed ASCT is generally poor, with a median overall survival of 2.4 years; for those who relapsed within a year of ASCT the number decreases to 1.2 years [6, 7].

Relapsed or refractory Hodgkin’s lymphoma not only has poor prognosis, but also brings heavy disease burden to patients. Studies have shown that as the number of HL relapses increases, the quality of life of patients decreases significantly [8]. Regarding treatment costs, it has been shown that the cost of second-line/third-line treatment is 3.5–2.7 times that of first-line treatment, and the average total treatment cost of transplant patients is 7–8 times higher [9]. Another study pointed out that for patients who failed first-line treatment, the cost of receiving treatment in the next 5 years is at least 14.3 times the average annual total cost of first-line treatment for disease control [10].

The availability of brentuximab vedotin (BV) offers a much-needed alternative for treating relapsed or refractory cHL (RRcHL). BV is an antibody drug conjugate that targets CD30-expressing malignant cells by binding CD30 on the surface. Targeted delivery of monomethyl auristatin E, the microtubule-disrupting agent, to CD30-expressing tumor cells is the primary mechanism of action [11, 12]. BV is approved for treating Adult patients with cHL after failure of ASCT or after the failure of at least two prior multi-agent chemotherapy regimens in patients who are not ASCT candidates by the U.S. Food and Drug Administration (FDA) and by the European Medicines Agency (EMA) [13, 14]. The approval is based on the efficacy and safety evidence from a pivotal multinational, open-label, phase II trial, where 102 RRcHL patients after ASCT were recruited and treated with BV, 1.8 mg/kg by intravenous infusion every 3 weeks (max. 16 cycles) (SG035-0003; NCT00848926) [15]. The results indicated that the overall response rate (ORR) was 75% with complete remission (CR) in 34% of patients [15]. It’s 5-year follow-up study showed that the estimated 5-year overall survival of 41% (95% CI: 31%, 51%) and the median OS was 40.5 months, based on a median follow-up of 35.1 months (range: 1.8–72.9 months) [16]. Furthermore, BV is also approved for post-ASCT consolidation for adult patients with cHL at high risk of relapse and progression. Most recently, the approval is extended for BV to be used in first-line settings in combination with chemotherapy in previously untreated advanced-stage cHL.

In China, BV was approved in 2020 for the treatment of adult patients with CD30-positive RRcHL [17]. The Chinese Society of Clinical Oncology (CSCO) guidelines also recommend BV for the treatment of RRcHL [18]. However, the economic value of BV compared with conventional therapy remains unknown. Thus, this present study aimed to conduct a cost-effectiveness analysis of BV versus conventional chemotherapy for treating RRcHL from a Chinese healthcare perspective, providing valuable insights for decision-making.

## Methods

### Expert survey

Due to the lack of local data, prior to the cost-effectiveness analysis, we conducted an expert survey to understand the treatment patterns and healthcare resource use associated with treating cHL in China. A total of 23 local experts, who worked in a tertiary hospital and treated individual lymphoma patients, were selected, respectively (see Appendix 1.). They were recruited from a range of locations, representing various economic development level and the number of lymphoma cases treated in different geographic areas in China. Specifically, information on treatment pathways, resource uses, and estimated costs for treatment was collected from each of the invited experts.

### Population

The model evaluates BV in adult patients with RRcHL (regardless of whether patients receive ASCT or not) [19].

### Comparators

Aligned with guidelines [1], surveyed local clinical experts indicated that in China the main treatment for patients with RRcHL is chemotherapy +/- radiotherapy with some patients followed by ASCT. The most commonly used chemotherapy regimens in China included GDP, GVD, ESHAP, BEACOPP, DHAP, and others. These high-dose chemotherapy regimens were here to represent a composite chemotherapy treatment, weighted by proportions of patients using each regimen, as the comparator.

### Model structure

The model represented a perspective of Chinese healthcare system, with a lifetime time horizon. A discount rate of 5% per annum is used for costs and health outcomes in line with China’s decision maker guidance [20].

A partitioned survival model in Microsoft Excel 2016, with a cycle length of 1 day, developed for the submission to Scottish Medicines Consortium (SMC) and later published in the literature [21], was adapted here. Briefly, three health states are included in the model: progression free (PF); post progression (PP); and death, and calculated by the PFS curve and overall survival (OS) curve. Costs and quality adjusted life years (QALYs) are accrued according to the proportion of patients in the PF and PP states over time. Costs and outcomes are evaluated on a daily basis for accuracy and simplicity.

Based on the expert survey, it was indicated that in China only a proportion of patients with adequate response after chemotherapy +/- radiotherapy received ASCT. It is estimated around 50% of patients with complete or partial response ultimately received ASCT. Furthermore, clinical experts also pointed out that the use of allogeneic stem cell transplants (alloSCT) in China is extremely limited due to its high costs, scarcity of donors, and hospital capability. Thus, it is recommended not to consider alloSCT in the current model.

Therefore, in order to compare BV versus chemotherapy +/- radiotherapy in treating RRcHL, two cohorts for each BV arm and chemotherapy+/- radiotherapy arm were built, representing patients with and without transplant after BV or chemotherapy, respectively. As suggested in the expert survey, it is assumed that the proportion of patients with adequate response after BV and followed by ASCT is the same as those receiving high-dose chemotherapy +/- radiotherapy. Thus, the proportion of patients who received ASCT was estimated as 29% and 10% in the BV arm and the chemotherapy +/- radiotherapy arm, respectively.

### Model input

### Clinical data

The C25010 trial, a single-arm, open-label, multi-center, phase II study, was conducted to examine the efficacy and safety of BV in Chinese patients, recruiting a total of 30 RRcHL patients [22]. BV was administrated as a single, 1.8 mg/kg IV infusion on Day 1 of each 3-week (up to 16 cycles) [22]. The C25010 trial provided the clinical input and baseline characteristics for the model. Additionally, the SG035-0003 trial, a multinational, open-label, phase II study of brentuximab vedotin with 102 RRcHL patients after ASCT, also provided the clinical input [16].

The sources of PFS and OS curves that populated the model are summarized in Table 1. As shown, in order to represent patients in the local setting, the PFS and OS curves of patients treated with BV only were based on the C25010 trial [22]. Due to the limited length of its follow-up (1.5 years), the data from the SG035-0003 trial was used to inform the following progression (the observed probability, up to 6.1 years) [16]. Due to the lack of the comparative arm in clicnial trials, for patients who received only chemotherapy +/- radiotherapy, the PFS curve was estimated from the participants in the SG035-0003 trial (self-control cases: the length from the last systemic therapy prior to BV until the starting of the BV treatment); the OS curve was based on one of the largest observational cohorts with 294 patients reported by Martinez and colleagues [23]. Finally, for patients with ASCT, the PFS, and OS were estimated from the study by Brockelmann et al. [24] based on a total of 1045 patients with ASCT from nine prospective trials – thus, one of the largest studies of RRcHL patients after ASCT. To reflcet the baseline characteristics of the local patietns, all PFS/OS curves retrived from the literature were adjusted by weighting the distribution of 0, 1, and ≥ 2 risk factors amongst patients in the C25010 trial. The risk factors included relapse within 3 months; Ann Arbor Stage IV disease; bulky disease; non-response to salvage chemotherapy; and ECOG > = 1. Beyond the observation period to lifetime, a constant risk was applied for PFS and OS curves, respectively, as those in the previous study [21]. It was reported that based on an European transplant registry data the probabilities of progression and death from ASCT patients were relatively constant from 30 to 40 months onwards, and therefore a constant probability of progression/death was assumed [21]. BV cohort after the trial period was assumed to use these constant risks and the assumption was tested in the sensitivity analyses.

**Table 1:** Sources of PFS and OS

Treatment	Outcomes	
	PFS	OS
Brentuximab vedotin (no SCT)	C25010 data (*n* = 30), 18 months; afterwards follow the progression rate of SG035-0003 trial (up to 6.1 years); then assume the same as that of ASCT cohort	C25010 data (*n* = 30), 18 months; afterwards follow the progression rate of SG035-0003 trial (up to 6.1 years); then assume the same as that of ASCT cohort
Chemotherapy (no SCT)	Self-control from SG035-0003 trial (*N* = 57), up to 3 years; afterwards assumes the same as that of ASCT cohort	Martinez 2013 (*n* = 294), 72 months, adjusting for the baseline risk distribution in C25010 trial; then assume the same as that of ASCT cohort
ASCT	Brockelmann 2017 (*n* = 1045), 86 months, adjusting for the baseline risk distribution in C25010 trial; afterwards assume a constant risk of progression (based on the finding reported in Parker et al. 2017).	Brockelmann 2017 (*n* = 1045), adjusting for the baseline risk distribution in C25010 trial; then assume a constant risk of death (based on the finding reported in Parker et al. 2017).

**Fig. 1:**
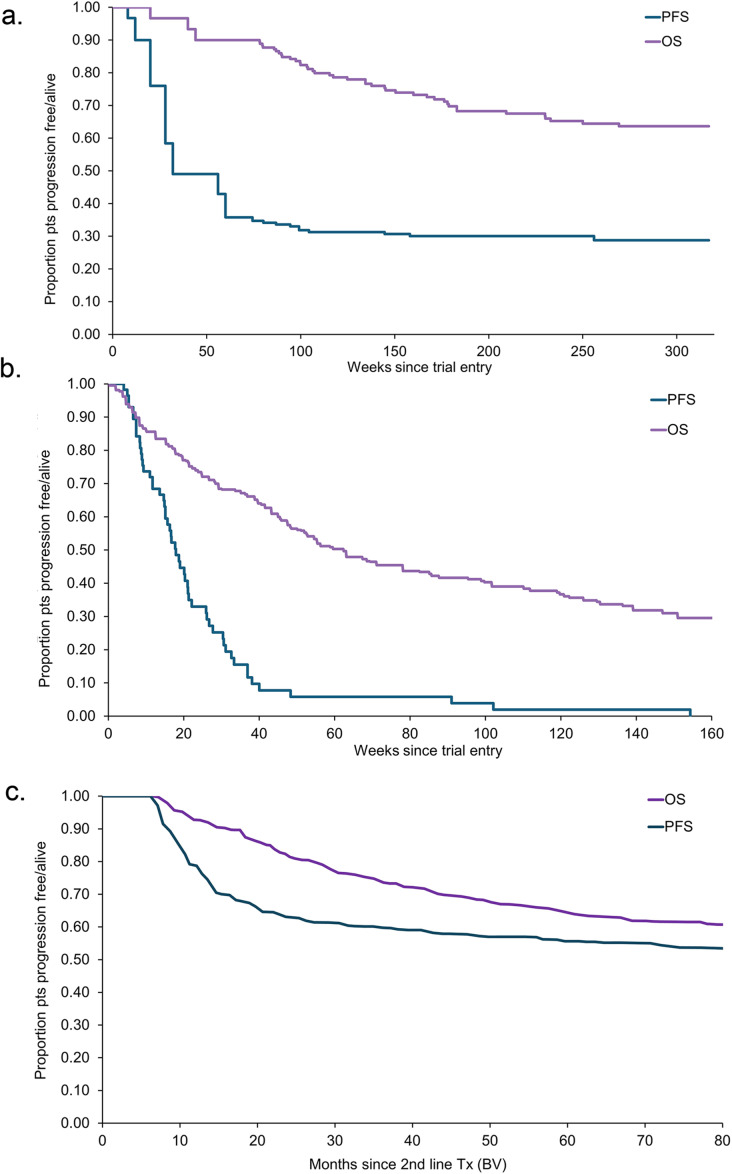
Progression-free survivor and overall survival inputs. PFS: Progression-free survivor; OS: overall survival; ASCT: autologous stem cell transplantation; BV: Brentuximab vedotin. (a) Brentuximab vedotin (based on the C25010 and SG035-0003 trial) (b) Chemotherapy (PFS from self-control case in the SG035-0003 trial; OS from Martinez 2013) (c) ASCT (from Brockelmann 2017. Assuming risk-free before receiving ASCT)

Furthermore, a hazard ratio of 0.5 was assumed to adjust for possible underestimation by using the data from the global trial SG035-0003 as more severe patients were involved in the global trial. This assumption was also examined in the sensitivity analysis. In the global trial SG035-0003, all patients received ASCT (100%) and averagely experienced 3.5 times of prior systemic treatments, whereas in the C25010 the numbers were 20% and 3.2, respectively. Thus, patients in the C25010 trial could be considered less severe than those in the global trial SG035-0003. The PFS and OS curves applied in the model are presented in Fig. 1a., 1 b. and 1 c., respectively.

### Resource utilization & costs

Resource utilization included drug acquisition and administration, concomitant medications, radiotherapy, ASCT, adverse events (AEs), and long-term follow-up. All unit costs [25] and estimated costs can be found in Table 2.

**Table 2:** Cost inputs

Chemotherapy regimen	Proportion [Expert survey]	Cost per cycle (21 days per cycle)	Source
GDP^1^	23%	$351 [¥2,421]	[28]
GVD^2^	13%	$2,060 [¥14,210]	[28]
ESHAP^3^	13%	$496 [¥3,422]	[28]
BEACOPP^4^	10%	$1,820 [¥12,555]	[28]
DHAP^5^	15%	$647 [¥4,460]	[28]
Others^6^	26%	$771 [¥5,317]	[28]
**Treatment**	**Risk of anti-fungal/viral/bacterial** [26, 27]**/[Expert survey]**	**Cost per week**	**Source**
Brentuximab vedotin	Low	$2 [¥13.0]	[28]
All chemotherapies	High	$21 [¥142.5]	[28]
**Treatment**	**Risk of emetics** [26, 27]**/[Expert survey]**	**Cost per week**	**Source**
All chemotherapies	High	$8 [¥54.7]	[28]
**Treatment**	**Proportion [Expert survey]**	**Package cost**	**Source**
Radiotherapy	30%	$8,151 [¥56,214]	[Expert survey]
ASCT	50% of patients with CR/PR	$23,608 [¥162,813]	[Expert survey]
**Item**	**Assumption [Expert survey]**	**Cost per unit**	**Source**
BV infusion	Costs associated with single infusion for BV	$30 [¥210]	[Expert survey]
Chemo infusion	Costs associated with infusion for chemotherapy	$146 [¥1010]	[Expert survey]
CT/PET scan		$203 [¥1,400]	[Expert survey]
Blood count		$2 [¥15]	[25]
Bio chemotherapy		$44 [¥300]	[25]
Consultation		$9 [¥60]	[25]

^1^GDP (gemcitabine, dexamethasone and cisplatin) [35]

^2^GVD (gemcitabine, vinorelbine and pegylated liposomal doxorubici) [36]

^3^ESHAP (etoposide, methylprednisolone, cytarabine and cisplatin) [37]

^4^BEACOPP (bleomycin, etoposide, doxorubicin, cyclophosphamide, vincristine, procarbazine and prednisolone) [38]

^5^DHAP (dexamethasone, cisplatin and cytarabine) [39]

^6^Otheres (AVD 8%, ABVD 31%, CMOPP 15%, GEMOX 15%, ICE 8%, IGE 15% and others 8% ) [40]

Assuming an average patient weight of 60 kg, it was estimated 2 BV vials per person per treatment cycle (per cycle 21 days). Thus, with the mean number of cycles observed in the SG035-0003 trial (9 cycles), it corresponds to a total cost of $18,797 (¥129,636) per course (assumed a full waste – no vial sharing). For the cohort of BV with ASCT, the number of brentuximab vedotin treatments is reduced to 7 cycles, following the opinions of clinical experts. For chemotherapy, the standard dosing for each regimen was obtained from the literature and confirmed by local experts (Table 2). The treatment cycle is assumed as 6 cycles for the cohort of chemotherapy; 5 cycles for the cohort of chemotherapy with ASCT. The proportion of patients in each regimen and the associated costs are presented in Table 2 Additionally, concomitant medications consisted of antiemetics treatment for brentuximab vedotin and all chemotherapies, and antifungal, antiviral, and antibacterial agents for all chemotherapies [26, 27]. Drug prices are taken from various open sources such as Yaozhi database (“药智网” in Chinese) [28].

Based on the survey with local experts, it was estimated that 30% of patients would receive radiotherapy ($8,151 [¥56,214) per person] in addition to their chemotherapy. According to the local experts, the total cost of ASCT was estimated as $23,608 (¥162,813), including the cost of acute adverse events or complications. The AEs included grade 1–2 nausea & vomiting, peripheral sensory neuropathy, and grade 3–4 thrombocytopenia, peripheral sensory neuropathy, neutropenia, leucopenia, anemia, and infection. The total cost of AEs was applied as a one-off cost and detailed costs are listed in Appendix 2.

Furthermore, follow-up was estimated based on clinical opinion and was stratified according to whether patients received ASCT and were on or off treatment. Details of resource uses associated with each follow-up period were depicted in Appendix 3. In the PP state, patients were assumed to experience a one-off cost of treatment on progression (equal to the chemotherapy acquisition and administration cost, as well as the total cost of AEs associated with standard chemotherapy). The long-term follow-up cost for post-progression patients was assumed to be equal to the cost for the on-treatment period for chemotherapy.

### Utility

Due to the lack of appropriate Chinese utility data, the utility data was adapted from the previous publication [21] Those utility data for each response category were sourced from a vignette study where utility values were elicited using the time trade-off method from 100 members of the general public in the UK [29], as shown in Table 3. In order to capture the impact of the different response categories on health-related quality of life, the utility level in the PFS health state was weighted according to the proportion of patients in each response category (i.e. complete response, partial response, and stable disease) for each comparator. The response data adapted by the model were also presented in Table 3 [15, 22, 30]. For patients in the PP health state, the corresponding utility for the progressed disease was assigned. For AEs, utility decrements, sourced from the literature, were combined with estimated event durations to generate a QALY decrement for each event. These AE utility decrements and durations were assumed to be the same as those used in the previous publication [21].

**Table 3:** Utility scores and response rates used to weight utility in PFS health state

Treatment	Utility scores Mean SD)	Brentuximab vedotin (%)	Chemotherapy +/- radiotherapy (%)	ASCT (%)
Complete response	0.91 (0.08)	6 (21%)	5 (15%)	56 (68%)
Partial response	0.79 (0.17)	15 (52%)	9 (27%)	19 (23%)
Stable disease	0.71 (0.20)	8 (28%)	19 (58%)	7 (9%)
Progressed disease	0.38 (0.28)	--	--	--
*Source*	Swinburn et al. 2015 [29]	The C25010 trial [22]	The global trial SG035-0003 [15]	Bierman 1996 * [30]

* The study reported response rates 3–6 months post-transplant. Thus, these response proportions are therefore assumed to apply to pre-progression patients from 100 days post-transplant until progression. Prior to 100 days post-transplant patients are assumed to experience the same response rates as on chemotherapy or brentuximab vedotin

### Sensitivity analysis

A range of sensitivity analyses were conducted, including testing the number of BV cycles, the proportion of patients receiving ASCT, the assumptions of PFS and OS curves after the clinical trial/observation period, utility score of patients in PFS state, chemotherapy cost, post-progress follow-up cost, ASCT cost, the hazard ratio of the local trial versus the global trial, the progression of self-control, local utility scores. Details can be seen in Appendix 4. A probabilistic sensitivity analysis was conducted, and distribution assumptions are described in Appendix 5.

## Results

### Long-term clinical outcomes

**Fig. 2:**
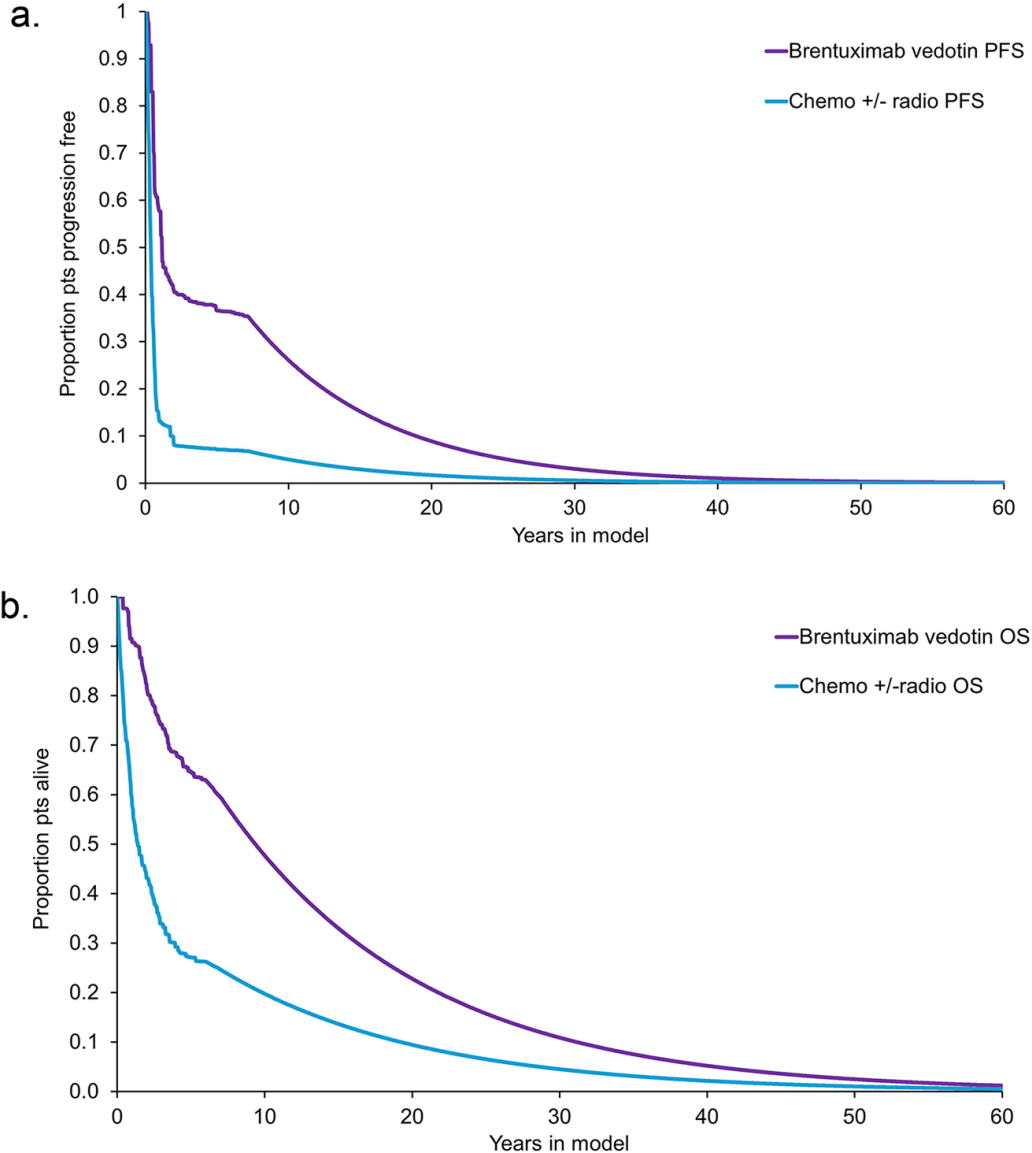
Predicted progression-free survivor and overall survival, by comparator. PFS: Progression-free survivor; OS: overall survival (a) Predicted progress-free survival (b) Predicted overall survival

Long-term outcomes predicted by the model are provided for each comparator below. PFS is presented in Fig. 2a. and OS in Fig. 2b. As shown in the figure, the PFS advantage of BV over the comparator presents over the modelling period. The data also show that BV has an OS advantage over the comparator throughout the entire period.

### QALYs

BV yields incremental QALYs of 2.97 vs. chemotherapy; this is driven by QALYs accrued in the PF health state which result from the greater mean PFS for BV (no SCT) compared to chemotherapy (no SCT). Disaggregated QALYs are presented for each comparator in Table 4.

### Costs

BV yields an incremental cost of $8,517 (¥58,739) vs. chemotherapy. This is driven by the cost of drug acquisition, offsetting the savings from administration, follow-up, and AEs treatments. Disaggregated costs by resource category are presented for BV vs. chemotherapy in Table 4.

**Table 4:** a. Disaggregated results of QALYs and costs

	QALYs			
Comparator	PF	PP	AEs	Total
Brentuximab vedotin	3.56	1.38	-0.02	4.92
Chemotherapy	0.95	1.03	-0.03	1.95
Incremental	2.61	0.35	0.01	2.97

b. Discounted costs, by comparator.

**Table:** 

Comparator	Acquisition	Admin. & concomitant	Follow-up	Adverse events	Total
Brentuximab vedotin	$24,084 [¥166,098]	$301 [¥2,073]	$15,703 [¥108,299]	$77 [¥528]	$40,165 [¥276,998]
Chemotherapy	$9,996 [¥68,939]	$2,195 [¥15,138]	$16,186 [¥111,631]	$3,270 [¥22,553]	$31,648 [¥218,260]
Incremental	$14,088 [¥97,160]	-$1,894 [-¥13,065]	-$483 [-¥3,332]	-$3,193 [-¥22,024]	$8,517 [¥58,739]

### ICERs

Thus, In the base case, the incremental cost-effectiveness ratio (ICER) for BV was $2,867 (¥19,774) per quality-adjusted life year (QALY) vs. chemotherapy +/- radiotherapy. BV was cost-effective under 1 time GDP ($12,426 [¥85,698] per QALY). The main driver of the incremental cost was the acquisition cost of BV treatment; the main driver of the Incremental QALYs was the advantageous PFS and OS of BV.

### Sensitivity analysis

**Fig. 3:**
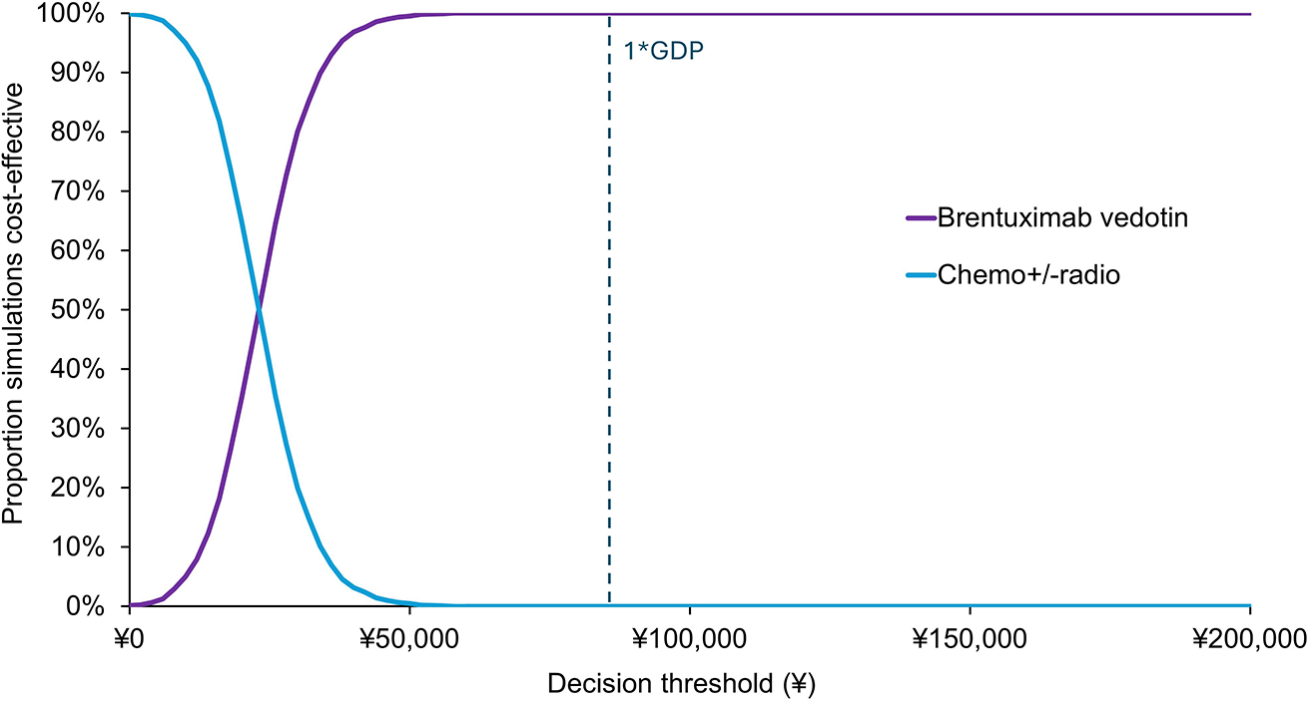
Cost-effectiveness acceptability curve

The results of the deterministic sensitivity analysis suggested that the ICER is most sensitive to the assumptions regarding the cycle number of BV treatment, the proportion of BV patients receiving ASCT, chemotherapy costs, and the cost of post-progression treatment. However, all sensitivity analysis results were still under a conventional decision threshold (1xGDP), suggesting the robustness of estimations. All sensitivity analysis results are shown in Appendix 4. The cost-effectiveness acceptability curve (CEAC) is presented in Fig. 3. At the 1xGDP threshold the probability of BV being the most cost-effectiveness was 100%.

## Discussion

This study investigated the cost-effectiveness of BV against chemotherapy +/- radiotherapy in treating RRcHL from the perspective of the Chinese healthcare system. The base case showed that BV yielded an incremental 2.97 QALYs gains under an incremental cost of $8,517 (¥58,739), which resulted in an ICER of $2,867 (¥19,774) per QALY gained. Thus, compared to chemotherapy +/- radiotherapy, BV is a cost-effective treatment under a 1xGDP threshold ($12,426 [¥85,698] per QALY). A series of deterministic sensitivity analyses showed that BV was cost-effective in all scenarios (all under 1xGDP threshold), whilst the probabilistic sensitivity analysis demonstrated that BV had 100% probability of being the cost-effective at the threshold .

The cost-effectiveness of BV versus chemotherapy +/- radiotherapy (with/without ASCT) in treating RRcHL has been demonstrated in many settings, including Scotland [21], Canada [31], Mexico, and Venezuela [32], and Sweden [33]. All studies except the one form the healthcare perspective in Canada indicated that BV is a cost-effective treatment option against chemotherapy comparators. The Canadian study showed that BV treatment generated more QALY gains but the ICER exceeded the Canadian threshold (above $100,000 per QALY).

The strength of the current study is to use the local trial C25010 data, representing real Chinese patients in the Chinese healthcare system. Together with the data collected from the local expert surveys, such as treatment pathways, resources use, and cost items, the model was able to reflect a real-world situation in China. For instance, the proportion of patients with ASCT and the very limited use of alloSCT all present a unique scenario in the setting of China. Thus, the result of the current model was highly relevant to the Chinese healthcare system and patients.

Furthermore, due to the lack of the BV data with long-term follow up, several assumptions were made in the current model, including using the data from the SG035-0003 trial and assuming a constant progression/death rate as the comparators for the long-term follow-up. These BV assumptions were tested extensively in the sensitivity analysis, such as the discrepancies between the C25010 and SG035-0003 trials (hazard ratio of 0.5, 0.7,0.8 and 1) and different progression/death rates for the long-term follow-up (hazard ratio of 1, 1.5 and 2, as well as the extrapolation of the existing data). All sensitivity analyses resulted in the same conclusion as the base case, suggesting that despite the various assumptions of BV, the model is relatively robust to these assumptions.

In the base case, the BV treatment cycle was based on the SG035-0003 trials (9 cycles). The average BV treatment cycle was 12 in the C25010 trial. The reason to choose the 9 cycles for the base case was that the number is aligned with the finding from a review of real-world evidence, suggesting the median number of cycles ranging from 4 to 8 [34]. The number of 9 cycles was also validated by the local clinicians. The sensitivity analysis showed that the use of 12 cycles increased the ICER to $4,361 [¥30,074] (51% increased comparing to the base case). However, it was still under 1 time GDP threshold.

There are a few uncertainties in the estimation of the model. First, the use of self-control data was a concern. Due to the lack of a comparator in both local and global trials (both were single-arm trials), participants’ prior chemotherapy before receiving BV (the PFS data of chemotherapy) was adapted. Such an approach might over- or under-estimate the outcome. For example, patients who died from the prior chemotherapy were not part of the trial (over-estimated); similarly, patients who improved from the prior chemotherapy were unlikely to be included in the sample (under-estimated). However, the results of sensitivity analysis suggested that the impact was limited.

Secondly, in order to capture the impact of the different response rates associated with different comparators on quality of life, the utility level in the progression-free state was weighted according to the proportion of patients in each response category (see Table 3). However, this approach did not capture the differential PFS periods associated with different response categories. The potential impact of this on the model results is explored as sensitivity analyses, by setting PF utility to that of the CR (to reflect the fact that the majority of patients remaining progression-free long term achieved a CR). The ICER decreased by 8% in this scenario.

Furthermore, all utility data were sourced from the literature and none of them represented the model population. Overall, the scarcity of utility data from the Chinese population is an issue for conducting economic modelling in this setting. Nevertheless, one local utility study was identified in the target literature review when conducting the model parameter search- a study collected the EQ-5D-5 L data from a total of 681 patients with Hodgkin’s lymphoma through an online survey. The result shows that the average EQ-5D-5 L utility score for patients with no progress, first-relapsed, relapsed > = 2 times, and responsive disease is 0.85, 0.86, 0.76, and 0.92, respectively. As the study population in this survey differs from our target population (relapsed or refractory) and does not provide all required utility data for the model, it was not used in the base case. Nevertheless, those utility scores were tested in the sensitivity analysis and suggested a similar result.

Future studies investigating the long-term follow-up of patients who received BV treatment would be needed. Such studies would help in understanding the long-term progression of the treatment and facilitate the estimation of the model. Additionally, more studies collecting local utility data from relevant populations should be encouraged given the scarcity of the data. Such data shall benefit all future economic modeling studies in China.

## Conclusion

In this cost-effectiveness analysis conducted from the perspective of the Chinese healthcare system, brentuximab vedotin is associated with an incremental cost-effectiveness ratio of $2,867 (¥19,774)/QALY compared to chemotherapy +/- radiotherapy. This estimate is under the range of threshold generally considered by decision makers in China (1xGDP: $12,426 [¥85,698]). Brentuximab vedotin can be considered a cost-effective treatment compared with chemotherapy +/- radiotherapy in treating relapsed or refractory classic Hodgkin’s lymphoma in China.

### Electronic supplementary material

Below is the link to the electronic supplementary material.

Supplementary Material 1

## Data Availability

Not applicable.
