# Supplementary material for: Cost-effectiveness of brentuximab vedotin compared with conventional chemotherapy for relapsed or refractory classic Hodgkin lymphoma in China
Source: Health Econ Rev. 2024 Jun 6;14:38. doi: 10.1186/s13561-024-00514-6 (PMC11155000; doi:10.1186/s13561-024-00514-6)
Supplement: Supplementary file 1 — Supplementary Material 1 [file 13561_2024_514_MOESM1_ESM.docx]

**Appendix 1. Participant list in the expert survey**

| No | City | Hospital |
| --- | --- | --- |
| 1 | Harbin | Institute of Hematology, Harbin First Hospital |
| 2 | Tianjin | Hematology Hospital of Chinese Academy of Sciences |
| 3 | Shanghai | Shanghai Zhongshan Hospital |
| 4 | Nanjing | People's Hospital of Jiangsu Province |
| 5 | Guangzhou | Cancer Hospital Affiliated to Sun Yat-sen University |
| 6 | Nanjing | People of Nanjing Hospital |
| 7 | Wuhan | Wuhan Tongji Hospital |
| 8 | Chongqing | Chongqing Cancer Hospital |
| 9 | Shanghai | Shanghai Ruijin Hospital |
| 10 | Tianjin | Tianjin Cancer Hospital |
| 11 | Beijing | PLA General Hospital |
| 12 | Urumqi | Xinjiang Medical University Tumor Hospital |
| 13 | Hefei | Anhui Cancer Hospital |
| 14 | Beijing | Peking Union Medical College Hospital |
| 15 | Changchun | First Affiliated Hospital of Jilin University |
| 16 | Guangzhou | Sun Yat-sen University Cancer Hospital |
| 17 | Zhengzhou | First Affiliated Hospital of Zhengzhou University |
| 18 | Guangzhou | People of Guangdong Province |
| 19 | Hangzhou | First Affiliated Hospital of Zhejiang University |
| 20 | Wuhan | Wuhan Union Hospital |
| 21 | Chengdu | West China Hospital |
| 22 | Fuzhou | Fujian Concord Hospital |
| 23 | Xiamen | First Affiliated Hospital of Xiamen University |

**Appendix 2. Frequency and costs of adverse events**

| **Grade** | **Adverse event** | **BV*** | **Chemotherapy*** | **Cost**** |
| --- | --- | --- | --- | --- |
| 1-2 | Nausea & vomiting | 21% | 6% | $0 [¥0] |
|  | Peripheral sensory neuropathy | 28% | 0% | $0 [¥0] |
| 3-4 | Nausea and vomiting | 0% | 16% | $58 [¥400] |
|  | Thrombocytopenia | 0% | 8% | $363 [¥2,500] |
|  | Peripheral sensory neuropathy | 0% | 0% | $53 [¥366] |
|  | Neutropenia | 18% | 18% | $363 [¥2,500] |
|  | Leukopenia | 5% | 19% | $247 [¥1,700] |
|  | Anaemia | 0% | 8% | $41 [¥280] |
|  | Infection | 0% | 50.1% | $6,282 [¥43,325] |

*BV based on the C25010 trial, weighted average of all chemotherapy regimens

**Source: from the expert survey, median price.

Appendix 3. Resource use during long-term follow-up

- Chemotherapy+/- radiotherapy or brentuximab vedotin)

| Time period (years) post-model entry | | Resource use per year | | | |
| --- | --- | --- | --- | --- | --- |
| Start of period | End of period | Outpatient visit (consultation) | Blood count | Biochemistry | CT/PET Scan |
| On-treatment | Off-treatment | 10.4 | 10.4 | 10.4 | 3 |
| Off-treatment | 2 | 4 | 4 | 4 | 0.5 |
| 3 | 5 | 2 | 2 | 2 | 0.33 |
| 5 | 20 | 1 | 0 | 0 | 0 |

- ASCT

| Time period (years) post-autogenetic transplant | | Resource use per year | | | |
| --- | --- | --- | --- | --- | --- |
| Start of period | End of period | Outpatient visit (consultation) | Blood count | Biochemistry | CT/PET Scan |
| 0 | 0.42 | 0 | 0 | 0 | 0 |
| 0.42 | 1 | 12 | 12 | 12 | 0.5 |
| 1 | 5 | 4 | 4 | 4 | 0.33 |
| 5 | 20 | 1 | 0 | 0 | 0 |

*The resource use in the period 0 to 0.42 (100 days) is set to zero as the total cost of these resource use is included in the total cost of ASCT.

Source: Expert survey

**Appendix 4. Deterministic sensitivity analysis and results**

| Deterministic sensitivity analysis | **Base case value** | **DSA value** | **ICER for brentuximab vedotin (per QALY)** | **% change relative to the base case** |
| --- | --- | --- | --- | --- |
| Base case | - | - | $2,867 [¥19,774] | - |
| BV treatment cycles | 9 cycles | 12 cycles | $4,361 [¥30,074] | 52% |
| Patients % in the BV arm receive ASCT | 50% | 25% | $2,351 [¥16,213] | -18% |
| Hazard ratio for PFS beyond the trial period for BV/chemo vs. ASCT | 1 | 1.5 | $3,097 [¥21,361] | 8% |
| Hazard ratio for PFS beyond the trial period for BV/chemo vs. ASCT | 1 | 2 | $3,241 [¥22,351] | 13% |
| Log-normal model (best available fit) for extrapolating PFS from the SG035-0003 trial | As ASCT | Extrapolation result | $3,171 [¥21,869] | 11% |
| Hazard ratio for OS beyond the trial period for BV/chemo vs. ASCT | 1 | 1.5 | $2,849 [¥19,649] | -1% |
| Hazard ratio for OS beyond the trial period for BV/chemo vs. ASCT | 1 | 2 | $2,838 [¥19,571] | -1% |
| Weibulll model (best available fit) for extrapolating OS from the Martinez 2013 data | As ASCT | Extrapolation result | $3,210 [¥22,141] | 12% |
| Hazard ratio for the local vs. global trial | 0.5 | 0.7 | $3,000 [¥20,691] | 5% |
| Hazard ratio for the local vs. global trial | 0.5 | 0.8 | $3,072 [¥21,183] | 7% |
| Hazard ratio for the local vs. global trial | 0.5 | 1 | $3,224 [¥22,237] | 12% |
| PFS of self-control | Original data | 20% | $2,934 [¥20,234] | 2% |
| PFS of self-control | Original data | 30% | $2,964 [¥20,442] | 3% |
| Utility score until progression | Based on the trial result | All CR utility | $2,634 [¥18,167] | -8% |
| Utility score of response category | UK-based score | China-based score | $2,787 [¥19,218] | -3% |
| Chemotherapy cost | Weighted average | Most expensive | $229 [¥1,577] | -92% |
| Chemotherapy cost | Weighted average | Least expensive | $4,184 [¥28,856] | 46% |
| Post-progression cost | $13,349 [¥92,062] | Increased by 100% | $2,171 [¥14,972] | -24% |
| Post-progression cost | $13,349 [¥92,062] | Set to zero | $3,564 [¥24,577] | 24% |
| ASCT cost | $23,608 [¥162,813] | +25% | $3,236 [¥22,315] | 13% |
| ASCT cost | $23,608 [¥162,813] | -25% | $2,499 [¥17,234] | -13% |

**Appendix 5. Distribution assumptions of probabilistic sensitivity analysis**

| **Parameter** | **Distribution** |
| --- | --- |
| Response rate | Dirichlet or beta (depending on reporting of data) |
| Kaplan Meier curves | Beta distribution was used to model probability of death in each time period of Kaplan Meier |
| Adverse events | Beta |
| Utilities (core health states) | Beta |
| Utility decrements (adverse events) | Beta |
| Duration of utility decrements | Log-Normal |
| Number of cycles received (all comparators) | Normal |
| Relative dose intensity (brentuximab vedotin) | Beta |
| Proportion of patients receiving each chemotherapy | Dirichlet |
| All expert opinion costs | Gamma |
| Other costs | Gamma |
| % patients proceeding to ASCT | Beta |
| Expert opinion estimates of proportion of patients receiving different resources | Beta |
| Expert opinion estimates of resource counts | Gamma |
